# Pharmacological Treatment of Presbyopia Using Pilocarpine 1.25% Eye Drops

**DOI:** 10.18502/jovr.v19i4.14578

**Published:** 2024-12-31

**Authors:** Seyed Abolghasem Mousavi, Zhale Rajavi, Hamideh Sabbaghi, Saeid Abdi, Nafeeseh Montazerin, Bahareh Kheiri, Azadeh Haseli-Mofrad, Kourosh Sheibani, Hemn Baghban Jaldian

**Affiliations:** ^1^Negah Aref Ophthalmic Research Center, Shahid Beheshti University of Medical Sciences, Tehran, Iran; ^2^Department of Ophthalmology, School of Medicine, Shahid Beheshti University of Medical Sciences, Tehran, Iran; ^3^Ophthalmic Epidemiology Research Center, Research Institute for Ophthalmology and Vision Science, Shahid Beheshti University of Medical Sciences, Tehran, Iran; ^4^Department of Optometry, School of Rehabilitation, Shahid Beheshti University of Medical Sciences, Tehran, Iran; ^5^Ophthalmic Research Center, Research Institute for Ophthalmology and Vision Science, Shahid Beheshti University of Medical Sciences, Tehran, Iran; ^6^Basir Eye Health Research Center, Tehran, Iran; ^8^Seyed Abolghasem Mousavi: https://orcid.org/0000-0001-9383-4378; ^9^Hamideh Sabbaghi: https://orcid.org/0000-0002-2627-7222; ^10^Zhale Rajavi: https://orcid.org/0000-0002-4078-3017

**Keywords:** Accommodation, Accommodation, Distance Vision, Eye Drops, Near Vision, Presbyopia

## Abstract

**Purpose:**

To assess the efficiency and safety of pilocarpine eye drop 1.25% analogue (IR- Pilo) in comparison with its original brand-name drug (Vuity).

**Methods:**

In this non-randomized comparative study, 75 patients with presbyopia aged 40 to 60 years were enrolled. The right eyes of these patients received either IR-Pilo (*n* = 45) or Vuity (*n* = 30) and their contralateral eyes served as controls. Refractive errors, distance best-corrected visual acuity (BCVA), near vision, amplitude of accommodation, pupil size, and intraocular pressure (IOP) were measured before and 1 to 2 hours after instillation of the eye drop.

**Results:**

The mean refractive error was stable, except for a small myopic shift in the Vuity group. There was no significant change in distance BCVA. Near vision improved significantly in both intervention groups (*P* < 0.001) with preference for IR-Pilo (4 vs 2.3). Furthermore, a higher amplitude of accommodation and pupil constriction occurred after instillation of both drops, with a higher effect associated with IR-Pilo. However, IOP did not change significantly post intervention.

**Conclusion:**

IR-Pilo and Vuity eye drops had comparable results; both were effective and led to stable distance vision and improved near vision. Both ophthalmic drugs were safe and none of them were associated with significant adverse effects.

##  INTRODUCTION

Presbyopia is one of the visual problems among individuals aged over 40 years old that reduces the quality of life and causes limitation in physical activities.^[1,2]^ Common therapeutic methods for this condition include optical modalities such as near glasses, bifocals or progressive spectacle lenses and monovision with contact lenses or refractive surgery.^[3]^ Near glasses are associated with a restricted field of vision, and contact lens wearers express concerns about dry eye symptoms.^[2]^ Meanwhile, higher accommodation and pupil constriction could enhance depth of focus and improve near vision, which could be induced by instilling different types of eye drops. Moreover, these drops could stimulate eye accommodation, increase flexibility of crystalline lens, and induce myopic shift.^[4,5,6,7]^ Pharmacological treatment of presbyopia is a noninvasive, safe, effective, fast, and accessible modality.^[4]^ Monotherapy with pilocarpine and combination therapy of pilocarpine and brimonidine or oxymetazoline or diclofenac are alternative methods for treating presbyopia.^[5,6]^ Specifically, pilocarpine 1.25% ophthalmic solution has been introduced as a safe pharmacological treatment for presbyopia.^[8,9]^ The effect of these drug modalities is initiated after 15 minutes of instillation, reaches its maximum in 1 hour, and lasts from 6 to 10 hours.^[5,6]^

In this regard, Benozzi et al explored the effect of a preservative-free combination therapy of pilocarpine and diclofenac (Benozz' method) in 910 patients with presbyopia, and followed them up for 1 to 8 years.^[10]^ Near vision significantly improved from 4.7 to 1.36 logMAR, while farsightedness remained stable without any discernible alteration. The reported complications such as headache, dizziness, eye redness, and dry eye were resolved spontaneously.^[10]^ Galeana confirmed the higher effectiveness of combination therapy with pilocarpine and brimonidine compared to monotherapy with either of these eye drops.^[11]^

In the current study, we compared the effectiveness of pilocarpine 1.25% eye drop manufactured by an Iranian Company (IR- Pilo) with its original brand-name drug (Vuity) for enhancing near vision among patients with presbyopia.

##  METHODS

This prospective non-randomized comparative study was performed on a total of 75 patients with presbyopia (*n* = 150 eyes) aged 40 to 60 years old. The patients had been admitted at the Negah Eye Hospital (Tehran, Iran) from March to September 2022. Prior to the study enrollment, an informed consent was obtained from all study participants. In one group, the right eyes of 45 patients were treated using IR-Pilo eye drop. In the other group, the right eyes of 30 patients received Vuity eye drop. In each group, the right eyes were considered as cases and the left eyes as controls.

The study procedures adhered to the Declaration of Helsinki and the study details were presented to the Ethics Committee of the Ophthalmic Research Center, Shahid Beheshti University of Medical Sciences, Tehran, Iran (approval number: IR.SBMU.ORC.REC.1401.005). The study protocol was also submitted to and approved by https://clinicaltrials.gov (identifier: NCT05564832).

Patients with the distance best-corrected visual acuity (BCVA) better than 0.3 logMAR and with the symptom of blurred vision at near distance ( < J3) were recruited for this study. Patients with amblyopia, cataract, refractive error > 1.00 D, corneal opacity, glaucoma, history of intraocular surgery, eye trauma, congenital pupil anomalies, and those with the history of headache and allergy to the eye drops were excluded from the study. Furthermore, we excluded patients with a history of systemic medications that adversely affected their accommodation amplitude.

Initially, distance BCVA was assessed using the Snellen E chart at a distance of 6 meters under daylight conditions. The refractive errors of all participants were measured by cycloplegic refraction, and patients with hyperopia > 1.00 D were excluded. In the next step, the near vision was checked by Jaeger chart at a distance of 33 cm under standard illumination.

Complete ophthalmic examinations were conducted to evaluate anterior and posterior ocular segments using slit-lamp biomicroscopy and indirect ophthalmoscopy, respectively. Then, the amplitude of accommodation was measured monocularly using the push-up techniques, and the result was recorded in diopters. The pupil size was checked and matched with the pupil hemisphere gauge ranging from 2 to 9 mm on the near chart under mesopic lighting conditions.

In the next step, the 1.25% pilocarpine IR-Pilo or Vuity eye drops were instilled into the right eye of each participant. All the mentioned examinations were repeated after 1 to 2 hours following eye drop instillation and patients were asked to inform us regarding any possible complications.

In the present study, comparison was conducted between case and control eyes in each group of IR-Pilo and Vuity eye drops. Furthermore, comparison was performed between eyes in the two intervention groups.

### IR-Pilo eye drop

One mL of pilocarpine 1.25% manufactured by Bakhtar Biochemistry Company (Iran) contains 12.5 mg pilocarpine hydrochloride as an active ingredient, equivalent to 1.06% pilocarpine free-base (10.6 mg). The preservative is 0.0075% benzalkonium chloride. Inactive ingredients in this ophthalmic solution are boric acid, sodium citrate dihydrate, sodium chloride, and purified water.

### Vuity eye drop

This drop (Vuity 1.25%, Allergan Company) contains 1.25% pilocarpine hydrochloride (12.5 mg/mL) as the active intergradient, equivalent to 1.06% pilocarpine free-base (10.6 mg/mL), and its preservative agent is 0.0075% benzalkonium chloride.

### Statistical analysis

We used mean, standard deviation, median and range, frequency and percentage to describe the data. Paired *t* test was used to assess changes within each group, and to evaluate the difference between the groups, independent *t* test was applied. Additionally, Generalized Estimating Equations (GEE) was used to explain any possible correlation between the eyes. All statistical analyses were performed using the SPSS version 25 (IBM Corp., Armonk, New York). *P*-values < 0.05 were considered statistically significant.

**Figure 1:**
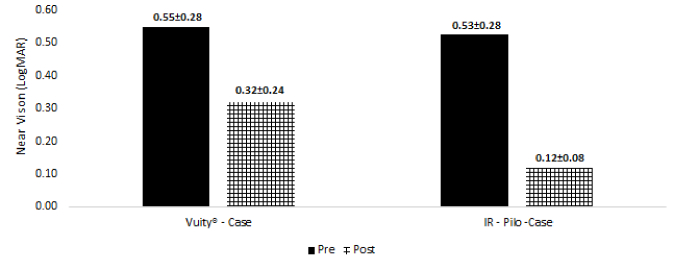
Near vision before and after administering the presbyopic drops (IR-Pilo and Vuity groups).

**Figure 2:**
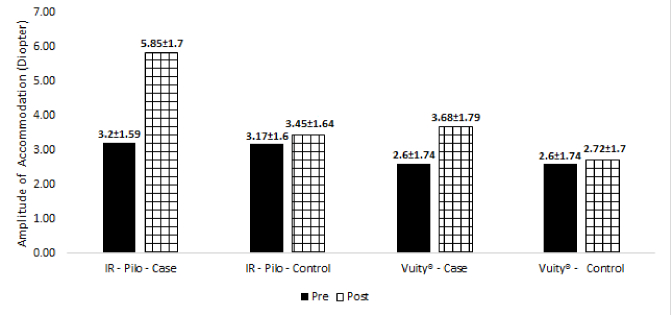
Amplitude of accommodation before and after administering the presbyopic drops (IR-Pilo and Vuity groups).

**Table 1:** Demographic characteristics of the presbyopic patients entering the study.

**Factors**		**Level**		**IR- Pilo**			***P*** **-value**	**VuityⓇ**			***P*** **-value**	***P*** **-value (between groups)**
				Case		**Control**		Case		**Control**		
Sex (%)		Male		17 (37.8%)		17 (37.8%)	> 0.999 ${\;}^{*}$	17 (56.7%)		17 (56.7%)	> 0.999 ${\;}^{*}$	0.274 ${\;}^{*}$
		Female		28 (62.2%)		28 (62.2%)		13 (43.3%)		13 (43.3%)		
												
Age (yrs)		Mean ± SD		48.04 ± 5.46		48.04 ± 5.46	> 0.999 ${\;}^{**}$	48.67 ± 5.56		48.67 ± 5.56	> 0.999 ${\;}^{**}$	0.892 ${\;}^{**}$
		Range (min to max)		47 (39 to 59)		47 (39 to 59)		48.5 (40 to 58)		48.5 (40 to 58)		
SD, standard deviation; yrs, years; min, minimum; max, maximum ${\;}^{*}$ *P*-value based on Chi-Square; ${\;}^{**}$ *P*-value based on *T*-test IR- Pilo: Presbyopia eye drop (1.25% pilocarpine) manufactured by Iran company; VuityⓇ: Presbyopia eye drop (1.25% pilocarpine) manufactured by USA company												

**Table 2:** Mean values of different indexes of refractive error in before and after instillation of presbyopia eye drops.

**Factors **			**IR- Pilo**				***P*** -value ${\;}^{*}$		**VuityⓇ**				***P*** -value ${\;}^{*}$		***P*** -value ${\;}^{**}$ (between cases)
			Case		**Control**				Case		**Control**				
Pre. Sphere (D)			0.47 ± 0.82		0.53 ± 0.95		0.753		0.48 ± 0.88		0.43 ± 0.85		0.824		0.943
Post. Sphere (D)			0.46 ± 1.3		0.54 ± 0.98		0.734		0.22 ± 0.7		0.29 ± 0.77		0.693		0.363
*P*-within ${\;}^{***}$			0.93		0.827				0.004		0.064				
Pre. Cylinder (D)			–0.59 ± 0.45		–0.48 ± 0.39		0.267		–0.5 ± 0.69		–0.68 ± 0.73		0.322		0.527
Post. Cylinder (D)			–0.51 ± 0.68		–0.54 ± 0.46		0.812		–0.55 ± 0.61		–0.42 ± 0.64		0.410		0.813
*P*-within			0.419		0.461				0.553		0.002				
Pre. SE (D)			0.23 ± 0.83		0.24 ± 0.95		0.963		0.14 ± 0.98		0.18 ± 0.91		0.865		0.692
Post. SE (D)			0.2 ± 1.16		0.27 ± 0.92		0.759		–0.06 ± 0.7		0.08 ± 0.78		0.463		0.283
*P*-within ${\;}^{***}$			0.78		0.556				0.033		0.210				
SE, spherical equivalent; D, diopter ${\;}^{*}$ *P*-value based on GEE; ${\;}^{**}$ *P*-value based on *T*-test; ${\;}^{***}$ *P*-value based on Paired *T*-test IR- Pilo: Presbyopia eye drop (1.25% pilocarpine) manufactured by Iran company; VuityⓇ: Presbyopia eye drop (1.25% pilocarpine) manufactured by USA company															

**Table 3:** Mean values of far and near visual acuities in before and after instillation of presbyopia eye drops.

**Factors**		**IR- Pilo**			***P*** -value ${\;}^{*}$		**VuityⓇ**			***P*** -value ${\;}^{*}$	***P*** -value ${\;}^{**}$ (between cases)	
		Case		**Control**			Case		**Control**		<@	
Pre. Far. BCVA (LogMAR)		0.01 ± 0.07		0.01 ± 0.06	0.321		0.0 ± 0.0		0.0 ± 0.01	0.921		0.313
Post. Far. BCVA (LogMAR)		0.0 ± 0.0		0.0 ± 0.0	0.330		0.0 ± 0.0		0.0 ± 0.01	0.921		> 0.999
*P*-within ${\;}^{***}$		> 0.999		> 0.999			> 0.999		> 0.999			
												
Pre. Near. UCVA (LogMAR)		0.53 ± 0.28		0.53 ± 0.29	0.989		0.55 ± 0.28		0.55 ± 0.29	0.935		0.769
Post. Near. UCVA (LogMAR)		0.12 ± 0.08		0.47 ± 0.29	0.003		0.32 ± 0.24		0.53 ± 0.28	< 0.001		< 0.001
*P*-within ${\;}^{***}$		< 0.001		0.074			< 0.001		0.017			
BCVA, best corrected visual acuity; UCVA, uncorrected visual acuity; LogMAR, logarithm minimum angle of resolution; SD, standard deviation ${\;}^{*}$ *P*-value based on GEE; ${\;}^{**}$ *P*-value based on *T*-test; ${\;}^{***}$ *P*-value based on Paired *T*-test IR- Pilo: Presbyopia eye drop (1.25% pilocarpine) manufactured by Iran company; VuityⓇ: Presbyopia eye drop (1.25% pilocarpine) manufactured by USA company												

**Table 4:** Mean values of accommodation, pupil size and IOP in before and after instillation of presbyopia eye drops.

**Factors**		**IR- Pilo**				***P*** -value ${\;}^{*}$		**VuityⓇ**				***P*** -value ${\;}^{*}$		***P*** -value ${\;}^{**}$ (between cases)
		Case		**Control**				Case		**Control**				
Pre. ACC (D)		3.2 ± 1.59		3.17 ± 1.6		0.926		2.6 ± 1.74		2.6 ± 1.74		1.000		0.127
Post. ACC (D)		5.85 ± 1.7		3.45 ± 1.64		0.000		3.68 ± 1.79		2.72 ± 1.7		0.039		0.000
*P*-within ${\;}^{***}$		0.000		0.177				0.000		0.184				
Pre. Pupil Diameter (mm)		2.68 ± 0.81		2.65 ± 0.84		0.869		2.78 ± 0.43		2.75 ± 0.41		0.759		0.505
Post. Pupil Diameter (mm)		1.23 ± 0.58		2.27 ± 0.96		0.000		1.68 ± 0.5		2.7 ± 0.41		0.000		0.001
*P*-within ${\;}^{***}$		0.000		0.006				0.000		0.083				
Pre. IOP (mmHg)		14.61 ± 2.36		14.42 ± 1.98		0.732		15.93 ± 2.55		16.07 ± 2.23		0.830		0.038
Post. IOP (mmHg)		14.13 ± 1.81		14.05 ± 1.75		0.857		15.8 ± 2.35		15.73 ± 2.27		0.912		0.003
*P*-within ${\;}^{***}$		0.076		0.048				0.564		0.077				
ACC., accommodation; IOP, intraocular pressure; D, diopter; mm, millimeter; mmHg, millimeter of mercury ${\;}^{*}$ *P*-value based on GEE; ${\;}^{**}$ *P*-value based on *T*-test; ${\;}^{***}$ *P*-value based on Paired *T*-test IR- Pilo: Presbyopia eye drop (1.25% pilocarpine) manufactured by Iran company; VuityⓇ: Presbyopia eye drop (1.25% pilocarpine) manufactured by USA company														

##  RESULTS

Table 1 presents the demographic characteristics of the current study population who received either IR-Pilo 1.25% or Vuity 1.25% eye drops. As shown, no significant difference was observed between the two groups.

Table 2 presents the mean values of spherical, cylindrical, and spherical equivalent refractions before and after instillation of IR-Pilo or Vuity eye drops. There was no significant difference in these refractive parameters between case and control eyes in the IR-Pilo group, whereas myopic shift was noted in spherical (*P *= 0.004) and spherical equivalent (*P *= 0.033) refraction after instillation of Vuity. Meanwhile, there was no difference in the pre- and post-treatment spherical and spherical equivalent between the IR-Pilo and Vuity groups.

In regard to visual acuity, no significant difference was found in distance BCVA before and after administering IR-Pilo and Vuity eye drops in each group and between the two groups. Furthermore, near VA significantly improved in both case groups (*P* < 0.001) compared with controls, however, this enhancement was more significant in the presbyopic eyes treated with IR-Pilo eye drop (4 lines vs 2.3 lines; *P* < 0.0001) [Table 3; Figure 1].

Table 4 shows the amplitude of accommodation, pupil diameter, and intraocular pressure (IOP) after administering the eye drops. As seen, a higher amplitude of accommodation (*P* < 0.001) [Figure 2] and pupil constriction (*P *= 0.001) were observed in eyes treated with IR-Pilo compared with Vuity; however, no significant difference was observed between the two groups in terms of IOP values.

Adverse effects including mild headache (20% and 23%), dry eye (9% and 16%), and dizziness (20% and 26%) were reported 2 hours after instilling IR-Pilo and Vuity drops, respectively. However, the complications were not severe and resolved spontaneously within one to two days after eye drop instillation.

##  DISCUSSION

In the present study, the mean refractive errors remained stable after pilocarpine therapy, although a small myopic shift (0.25 D) was observed after administering Vuity. No significant changes in distance BCVA were detected, yet near VA and amplitude of accommodation significantly improved after administering both presbyopic eye drops, with higher improvement being achieved by IR-Pilo drop. In addition, a higher pupil constriction was noted after instilling IR-Pilo eye drops, yet IOP did not differ after administering the ophthalmic solutions.

Benozzi et al^[12]^ investigated the effectiveness of the combination therapy of pilocarpine and diclofenac without preservative agents (Benozzi Method) on 148 patients with presbyopia. They observed that while the uncorrected VA at far distance did not change, the near VA improved from J ${\;}_{8}$ - J ${\;}_{3}$ to J ${\;}_{3}$ - J ${\;}_{1}$ (equal to two to six lines) within the follow-up period between 2 to 10 years. Their results are in line with our findings even in short follow-ups. Similar to the present study, the authors concluded that pilocarpine therapy could be safe and effective for treating presbyopia.

In another study, Benozzi et al^[10]^ aimed to determine the safety and efficacy of the Benozzi method on 910 patients with presbyopia with uncorrected distance VA better than 0.1 logMAR and the near VA of worse than J ${\;}_{2}$ . After one year, changes in uncorrected distance VA were not significant, while the near VA improved from 4.74 ± 1.5 to 1.36 ± 0.48 logMAR based on Jaeger chart (equal to 3.5 lines). The mean of near visual improvement by IR-Pilo drop in our study was similar to their study. While near VA improvement was slightly less (2.3 lines) in the Vuity group, it was still within the range reported by Benozzi et al. This difference could be attributed to the distinct method used to measure VA, which was monocular in our study and binocular in that study.

In another study, the effectiveness of Vuity was reported and significant improvement in low-light corrected near vision occurred without losing more than one line of corrected distance vision at 3 hours and 6 hours after eye drop instillation.^[9]^

The prospective interventional study by Vargas et al^[13]^ on 117 patients with presbyopia showed that pilocarpine eye drop could improve near VA from 0.35 to 0.16 logMAR (*P* < 0.0001) 2 hours after administering the drop. Overall, near vision improved by two lines in 92.3% of patients. The outcomes reported in that study are similar to our findings in terms of age, evaluation time (2 hours), and degree of near vision improvement in the Vuity group.

The possible mechanisms for the improvement of near VA after instillation of pilocarpine eye drop have been discussed by Montés-Micó et al.^[6]^ They reported that most studies have measured visual acuity at far and near distances under mesopic conditions, while induced pupil constriction might influence vision under both mesopic and scotopic conditions. Additionally, higher depth of focus and myopic shift are the other etiologies of near vision improvement after pilocarpine therapy. In the present study, a small myopic shift was noted in the Vuity group.

Price et al^[14]^ studied the efficacy of the combination therapy of 0 to 1.5% pilocarpine and 0.5 to 0.125% oxymetazoline in order to reduce the headache following the drug usage. They found that the drug's effect initiated after 15 minutes of instillation and maximum effectiveness was obtained within 1 hour. No significant difference was noted in distance VA, while near VA improved from day 1 to day 14 and it was maintained until day 28. It was also concluded that 1.25% pilocarpine was the best dosage for treating presbyopia. The findings of our study are comparable with this study in terms of the short-term effect of these drops and the consequent increased near VA and stable distance VA.

Vargas et al^[13]^ investigated the safety and efficacy of pilocarpine for treating presbyopia. The authors conducted comprehensive ophthalmic assessments, including the following measurements: BCVA at both far and near distances, refractive errors, pupil diameter, Schirmer test, endothelial cell count, IOP, anterior chamber depth as well as keratometry and pachymetry. These assessments were performed at 30-minute intervals and were repeated every hour for 5 hours followed by 1 week and 1 month. The results showed that all patients were satisfied with the improvement of near vision (between one and three lines) and no case reported blurry vision at far distance neither in monocular nor binocular conditions. A maximum myopic shift of –0.50 D was observed, but it resolved after 4 hours. Additionally, no effects were identified on tear layer, endothelial cells, and corneal pachymetry. Also, IOP was reduced by 2 mmHg after 5 hours of administering the eye drop. Our findings are in line with this study in terms of both distance and near vision and the small myopic shift we noted in the Vuity group.

Headache, dizziness, red eye, and dry eye were the most common symptoms during the 2 hours of our study, but they spontaneously resolved within one to two days after eye drop instillation.

There are some points that should be considered when evaluating the results of the current study. In most existing studies on the pharmacological treatment of presbyopia, both eyes have received the eye drop. However, in the present study, only the right eye received pilocarpine eye drop (either IR-Pilo or Vuity) and the fellow eye was considered as the control. While the presence of a matched control group is a strength of this study, it could also be a limitation due to reporting a lower monocular VA compared to binocular VA in other studies.

Most previous studies have included patients with presbyopia who have emmetropia and uncorrected VA of 0.1 logMAR at distance and near VA worse than J2, while we included patients with distance BCVA better than 0.3 logMAR and near VA less than J3. This could be considered a strength of the present study, offering higher external validity.

In the present study, the mean improvement of near vision was 4 and 2.3 lines in patients receiving IR-Pilo and Vuity, respectively, which is in line with other studies that report a mean increase of near vision from two to six lines. Besides, the amplitude of accommodation was greater in patients treated by IR-Pilo compared to Vuity (2.50 D vs 1.00 D), although the latter resulted in a better near vision. It might be associated with transient headache.

Better near visual acuity due to enhanced depth of focus following pupil constriction, having matched controls, and high external validity due to including cases with a wider range of reduced near vision are the strengths of our study. A relatively small sample size, applying the pilocarpine drops unilaterally, short follow-up period, and the unpleasant sight of constricted pupil in cases with light iris color were the limitations of the present study. Finally, although spherical change was not significant between the two groups after using each drop (*P *= 0.363), and there was a myopic shift of 0.25 D in patients treated by Vuity, near vision was higher in the IR-Pilo group. These findings could be explained by considering the higher pupil constriction and amplitude of accommodation as well as enhanced depth of focus in the IR-Pilo group, which could have offset the small myopic shift induced by Vuity.

In summary, based on the results of the current study, IR-Pilo and Vuity eye drops, particularly the former, were effective for presbyopia and showed stable distance vision and improved near vision. Both ophthalmic drugs were safe and neither was associated with any significant adverse effects. It is recommended that further studies be conducted with larger sample size and longer follow-ups and bilateral instillation of presbyopia drops.

##  Financial Support and Sponsorship

None.

##  Conflicts of Interest

None.
